# Multiple Hepatic Lipoma: A Case Report of Captive Hill Mynah with Iron Storage Disease

**DOI:** 10.3390/vetsci10100626

**Published:** 2023-10-19

**Authors:** Seoungw-Woo Lee, Soong-Hee Youn, Jin-Kyu Park

**Affiliations:** 1 Department of Veterinary Pathology, College of Veterinary Medicine, Kyungpook National University, Daegu 41566, Republic of Korea; pyrk2000@dgist.ac.kr (S.-W.L.); hapysh3@naver.com (S.-H.Y.); 2 Division of Biotechnology, DGIST, Daegu 42988, Republic of Korea; 3 Samsung Everland Zoological Garden, Yong-in 17023, Republic of Korea

**Keywords:** captive animal, hill mynah, iron storage disease, lipoma

## Abstract

**Simple Summary:**

The lipoma is a benign tumor consisting of mature adipocytes. Although lipomas can arise anywhere on the body, hepatic lipomas are rarely observed; thus, only a few hepatic lipoma cases are documented in veterinary medicine. According to previous research, the majority of hepatic lipomas are generally observed as solitary nodular structures. However, recently, multiple hepatic lipomas were found in a captive hill mynah. In the present case, the liver tissue from the bird exhibited not only multiple hepatic lipomas, but also excessive iron accumulation. To the best knowledge of the authors, this is the first report of multiple hepatic lipomas accompanying ISD, and herein, these unique pathological findings are reported.

**Abstract:**

The present case describes multiple hepatic lipomas in a common hill mynah (*Gracula religiosa).* A 21-year-old female captive common hill mynah died without any notable clinical symptoms. An autopsy and histopathological examinations were conducted to determine the exact cause of death. On gross observation, the liver demonstrated a yellowish white surface color and multiple nodules indicating neoplastic lesions. Histopathological assessment found that the nodules purely comprised mature adipocytes. Furthermore, the liver exhibited an excessive accumulation of iron in hepatocytes and Kupffer cells and the infiltration of chronic inflammatory cells, suggesting iron storage disease (ISD). Based on the results, the present case was diagnosed as multiple hepatic lipomas with ISD. To the authors’ best knowledge, multiple hepatic lipomas accompanying ISD lesions have not been reported previously. Hence, the present case is the first case report of hepatic multiple lipomas with ISD in veterinary medicine.

## 1. Introduction

The hill mynah (*Gracula religiosa*) is a bird belonging to the family *Sternidae* of the order Passeriformes. It has an average length of 27–30 cm and an approximate body weight of 400 g. Its body is characterized by a yellow crest that starts from the sides of its eyes to the neck, pale blue-green tones of black feathers on the rest of the body, and a white spot on each of the third to the ninth primary wing feathers [1]. It prefers highland rainforests and moist environments and is mostly found in jungles, evergreen forests, and forest margins. West India, southern China, Indochina, Thailand, Malaysia, and the Philippines are among its natural habitats [2]. However, owing to the destruction of its natural habitats and the indiscriminate poaching in China, Indonesia, Malaysia, and Nepal, the natural population of the hill mynah is constantly decreasing; thus, most of them are found in zoos and breeding farms rather than in their natural habitats. Therefore, the health management of hill mynahs in zoos is crucial for species conservation [3,4].

Hepatic lipoma is a tumor composed of well-differentiated mature adipocytes, and it is usually diagnosed through incidental findings because of its asymptomatic features [5]. According to previous studies, hepatic lipoma is generally rare, and in veterinary medicine, few cases of hepatic lipoma have been reported. Previously, Stimmelmayr et al. (2017) and Rezaie et al. (2015) reported hepatic lipoma in bowhead whales and camels [6,7]. In companion animals, de la Vega et al. (2023) reported hepatic lipoma in dogs, and in birds, Latimer et al. (1995) and Musa et al. (2019) documented lipomatous tumor in exotic birds and chickens [8,9,10]. However, despite efforts exerted to understand the underlying mechanisms of these tumors, the precise origin and etiology of hepatic lipoma remains unclear.

Iron is a vital nutrient required by various physiological processes in living organisms [11]. However, the excessive accumulation of iron can initiate damage to cells via free-radical generation [12]. Iron storage disease (ISD) is defined as a disorder caused by an excessive burden of iron in the body [13]. According to previous reports, ISD frequently occurs in many captive animal species and can provoke systemic organ failure, chronic metabolic disorders, and even cancer [14,15]. However, tumorous lesions accompanying ISD have rarely been reported in hill mynah species, despite its high prevalence. Recently, we found multiple hepatic lipomas accompanying ISD in a captive hill mynah. In the microscopic findings, the tissue sample from the bird’s liver not only showed multiple lipomas, but also exhibited chronic inflammatory lesions caused by excessive iron accumulation. Considering most reports of hepatic lipoma involve a benign, solitary nodule, we suggest that there is close association between multiple hepatic lipomas and ISD. To the authors’ best knowledge, this is the first report of spontaneous multiple hepatic lipomas accompanying ISD in veterinary medicine, and herein, we report on this unique pathological finding.

## 2. Case Presentation

A 21-year-old female hill mynah that had exceeded the average lifespan was being managed at old age. Regular weighing was performed; however, it unexpectedly died after manifesting weakness and anorexia. The hill mynah was fed a 10 g commercial parrot diet, 10 g papaya once weekly, two mealworms (twice a week), and a low-iron-containing toucan diet (iron ≤ 25–50 mg/kg) to prevent excessive iron accumulation in the animal [16]. Serum iron levels were measured through blood biochemistry once annually, with 101.0 and 99 μg/dL being recorded in 2019 and 2020, respectively, which were not higher than the iron levels of other subjects (Table 1). Additionally, the blood iron levels in the birds were similar to other previous reports describing blood iron levels in normal mynahs [17].

**Figure 1:**
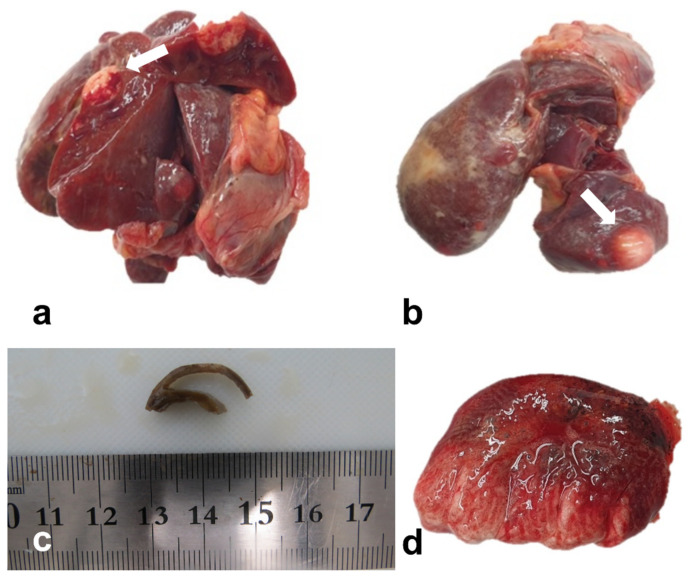
Parenchymal organ samples of the hill mynah. (**a**,**b**): The liver had a yellow-whitish nodule (white arrow). (**c**): The formalin-fixed heart showed a thin myocardial wall. (**d**): A brown surface color was noted in the lung.

The animal showed no notable clinical symptoms except for a minimal decline in body weight. A necropsy was immediately conducted to unveil the exact cause of the death. The bird was emaciated, and the animal’s body weight was approximately 320 g. Other specific external lesions were not found. Grossly, the liver tissue sample showed a yellowish surface color and multifocal nodules suggesting spontaneous tumorous lesions (Figure 1a,b). The heart had a thin myocardial wall (Figure 1c), and the lung demonstrated a reddish brown surface color, suggesting an edematous lesion (Figure 1d).

**Figure 2:**
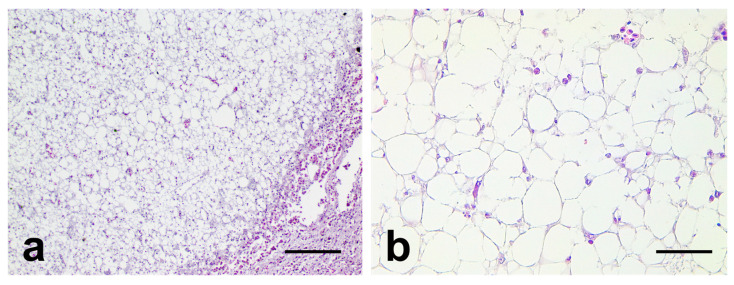
Representative microscopic images of the lipomatous nodules. (**a**,**b**): Representative images of nodules located in the liver. The nodules mainly comprised adipocyte-like cells. Bar = (**a**): 200 μm and (**b**): 50 μm.

Any other remarkable findings were not noted in the parenchymal organs, and for histopathological assessment, the liver, heart, and lung tissue samples were fixed in 10% neutral buffered formalin and embedded in paraffin. The paraffin blocks were sectioned at 5 μm thick, and stained with hematoxylin and eosin (H&E), Masson trichrome (MT), and Prussian blue staining for histopathological examination. In the microscopic observation, the multifocal hepatic nodules were found to mainly consist of well-differentiated large polygonal cells characterized by a clear cytoplasm and small eccentric nuclei with no hematopoietic cells (Figure 2a,b).

Based on these histopathological findings, angiomyolipoma, macrovesicular fatty change, and lipoma were considered as a differential diagnosis. Hepatic angiomyolipoma is a tumor originating from the perivascular epithelioid cells of the liver, which is a rare and benign neoplasm in humans. It mainly comprises a mixture of various mesenchymal cells, such as fibroblasts, smooth muscles cells, and adipocytes, according to previous studies [18]. However, in the current case, fibroblasts and smooth muscle cells were not observed in the sample, and thus, hepatic angiomyolipoma was ruled out. Next, macrovesicular fatty change was considered. Although macrovesicular fatty change is also characterized by enlarged clear hepatocytes mimicking mature adipocytes, it is generally known as a diffuse pathologic process [19]. Thus, macrovesicular fatty change was also ruled out. Finally, a hepatic lipoma was considered. According to previous research, a hepatic lipoma is a tumor composed of fully differentiated adipocytes, and it can occur at any site, even in locations where the adipose tissues are not normally present [20]. The hepatic nodules were mainly composed of polygonal cells characterized by eccentric nuclei and a large clear cytoplasm, and normal hepatic architectures such as hepatic cords, veins, and arteries were not observed in the nodules. Thus, the enlarged polygonal cells were identified as a mature adipocyte, and the neoplastic nodules were diagnosed as multiple hepatic lipomas.

**Figure 3:**
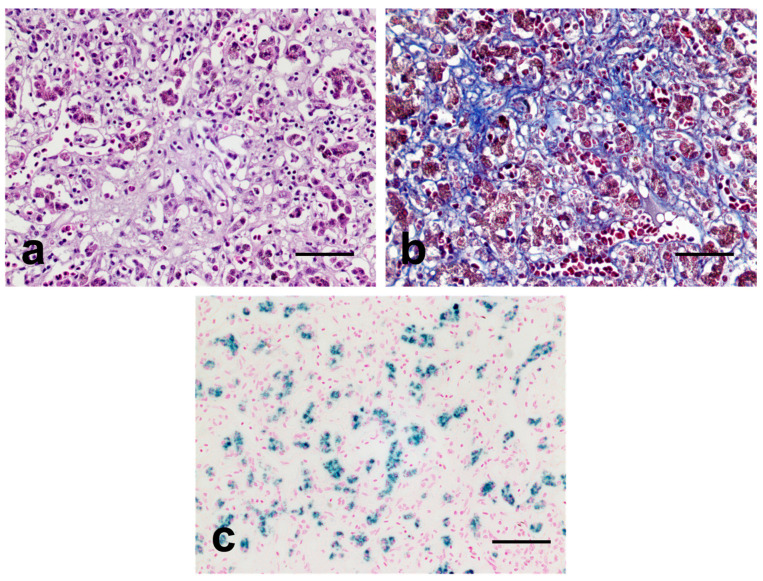
Representative microscopic images of the liver. (**a**): Hematoxylin and eosin (H&E) staining images of the liver tissue. Note the infiltrations of chronic inflammatory cells and fibrotic lesions. (**b**): Masson trichrome (MT) staining image of the liver tissue. Note the prominent positive responses for MT staining in liver parenchyma. Fibrous tissue is highlighted in blue on MT stain. (**c**): Prussian blue staining images of the liver. Hepatocytes and Kupffer cells displayed a strong positive response for Prussian blue staining. The iron granules are highlighted in blue on Prussian blue stain. Bar = 50 μm.

Following histopathological diagnosis, it was also revealed that the liver tissue sample also displayed infiltrations of chronic inflammatory cells, necrosis, and severe fibrotic lesions, indicating chronic liver injuries (Figure 3a,b). Moreover, hepatocytes and Kupffer cells were noted, with numerous dark-brown cytoplasmic granules suggesting excessive iron accumulation. Thereafter, these granules showed a strong positive response for iron-specific (Prussian blue) staining (Figure 3c). These results clearly suggested the presence of ISD characterized by the excessive accumulation of iron or ferritin in the liver [17,21]. Considering that ISD can cause chronic lesions, including ischemia and fibrosis to the liver via free-radical generation in cells, it is suggested that ISD-mediated prolonged liver damage might have been the primary cause of death in the bird [21,22].

**Figure 4:**
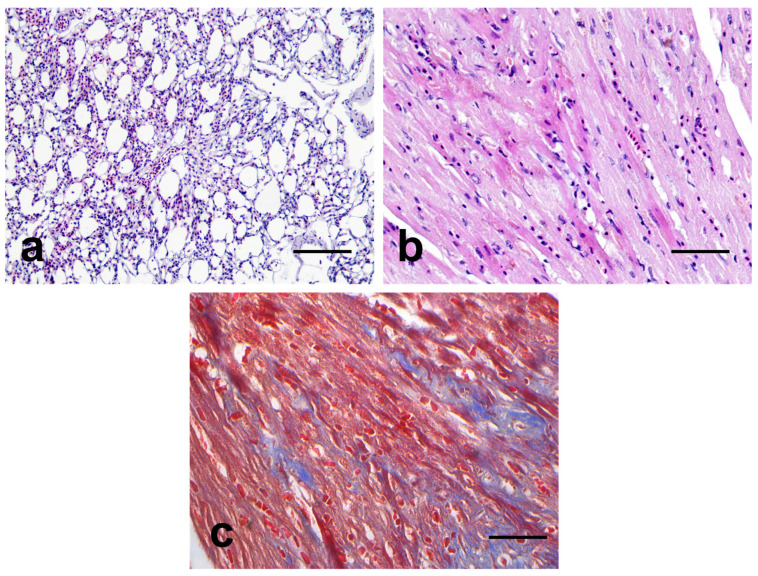
Representative microscopic images of the lung and heart. (**a**): H and E images of the lung. The lung exhibited mild emphysema and congestion. (**b**,**c**): H and E and MT images of the heart. The heart showed mild fibrosis in the cardiac muscle. Note the weak positive responses for MT staining in the heart. Fibrous tissue is highlighted in blue on MT stain. Bar = (**a**): 200 μm, (**b**,**c**): 50 μm.

Other parenchymal organs, such as the lung and heart, showed age-related lesions. In the lungs, the normal structures were generally well retained, except for hyperemia and weak emphysematous lesions (Figure 4a). In the heart, weak fatty degeneration and cardiomyocyte atrophy with mild fibrosis were observed (Figure 4b,c).

The possibility of an infectious disease was also considered, and many special stains including Gram and Macchiavello staining were performed. The results of special stains for infectious entities were all negative; thus, we could confirm the absence of infectious agents, such as bacteria, parasites, and viruses, in the body of the bird via histopathological assessment. Finally, the present case was diagnosed as multiple hepatic lipomas with ISD. High-resolution histopathological images are presented in the Supplementary Materials (Supplementary Figures S1–S8).

## 3. Discussion

According to previous studies, a hepatic lipoma diagnosis can often be challenging, as it mimics the imaging appearance and histopathological features of other diseases [23,24]. When considering the clinical importance of a tumorous lesion in the liver, understanding the differences between a benign hepatic lipoma and other cancerous diseases is important both in clinical practice and in pathological studies. In the present study, we report multiple hepatic lipomas found in veterinary medicine, and we conclude that it can be utilized as valuable knowledge for diagnosing tumorous lesions in the liver. According to previous studies, most hepatic lipomas present with a benign solitary nodular structure [25,26,27,28,29]. However, in the present case, the lipoma showed a multifocal growth pattern. Thus, an important question is posed: how do multiple lipomas simultaneously occur without any malignant lesions? Although the precise etiology of hepatic lipomas remains unclear, many researchers have reported that the excessive aggregation of triglycerides in the liver might be a primary cause [5]. Consistently, it is known that the prevalence of hepatic lipoma is closely related to lipid metabolism disorders, such as dyslipidemia and nonalcoholic fatty liver disease (NAFLD) [16]. In the present case, the birds demonstrated multifocal lipomatous nodules, indicating an increased upregulation of triglycerides in the liver. However, during gross observation, the bird was emaciated; thus, NAFLD caused by excessive nutrient uptake was ruled out as a possible cause. Then, we focused on the lesions caused by ISD and ISD-mediated lipid metabolic disorder. Recently, Li et al. (2023) reported that a high serum iron concentration is positively correlated with serum lipid levels, and Rajpathak et al. (2009) contend that an increase in serum iron levels is an important risk factor for dyslipidemia [30,31]. When considering this prior research and severe hepatic ISD lesions in birds, it is assumed that ISD-mediated lipid dysregulation could be a primary cause of multiple occurrences of benign hepatic lipomas. Altogether, the present case reveals a case of multiple hepatic lipomas, possibly secondary to an ISD. Considering that many lipoma cases are incidentally detected due to their asymptomatic presentation, it is anticipated that there could be many unreported lipoma cases in captive hill mynahs [5]. Therefore, in the present case, we showed that hepatic lipoma needs to be considered as a potential ISD-mediated lesion.

According to previous studies, ISD can be a main cause of death in birds, and it is closely associated with diseases of the liver and the heart [32,33]. Thus, in many zoos, phlebotomy and blood tests are routinely performed at least once every 3–4 months to prevent ISD. However, preventing the occurrence of ISD is challenging [21]. Moreover, prior research also reported that the hill mynah species can be easily affected by ISD owing to its genetic predisposition [34]. Accordingly, in the present case, the bird manifested with both severe ISD lesions and a low-to-average serum iron concentration. Considering that the bird was fed with a low-iron diet to prevent iron accumulation, these data suggest that ISD is challenging to manage using only low-iron diet feeding and serum biochemistry. Therefore, ISD is an important metabolic disorder in captive animals, and the present case contributes novel insight regarding the risk of ISD in captive animals.

## 4. Conclusions

The present case was diagnosed with multiple hepatic lipomas accompanied by an ISD. According to the authors’ best knowledge, the present case is the first report on spontaneous multiple hepatic lipomas accompanying ISD in veterinary medicine. We believe that the present case can foster a better understanding of hepatic lipomas and ISD in the captive animal. Moreover, considering that ISD in hill mynahs has many similarities to human iron-mediated disorders, we anticipate that the present case can also be useful for understanding ISD and ISD-related lesions in many other species, including humans [17].

## Figures and Tables

**Table 1:** Blood iron levels in the captive hill mynahs.

Case	Age	Sex	Date (dd.mm.yy)	Blood Iron Level (μg/dL)
Control bird 1	10	Unknown	28.10.19	134
			10.11.20	71
			11.03.21	101
Control bird 2	15	Female	29.10.19	137
Control bird 3	15	Male	29.10.19	116
			10.11.20	93
Control bird 4	15	Male	29.10.19	106
			10.11.20	76
Subject 5 *	21	Female	07.07.19	101
			10.11.20	99

* Defines bird in the present case.

## Data Availability

Data will be available on request.

## Supplementary Materials

The following supporting information can be downloaded at: https://www.mdpi.com/article/10.3390/vetsci10100626/s1, Figures S1–S8: Original high-resolution microscopic images from the present case.
