# Supplementary figures and images for: Multiple Hepatic Lipoma: A Case Report of Captive Hill Mynah with Iron Storage Disease
Source: Vet Sci. 2023 Oct 19;10(10):626. doi: 10.3390/vetsci10100626 (PMC10611273; doi:10.3390/vetsci10100626)

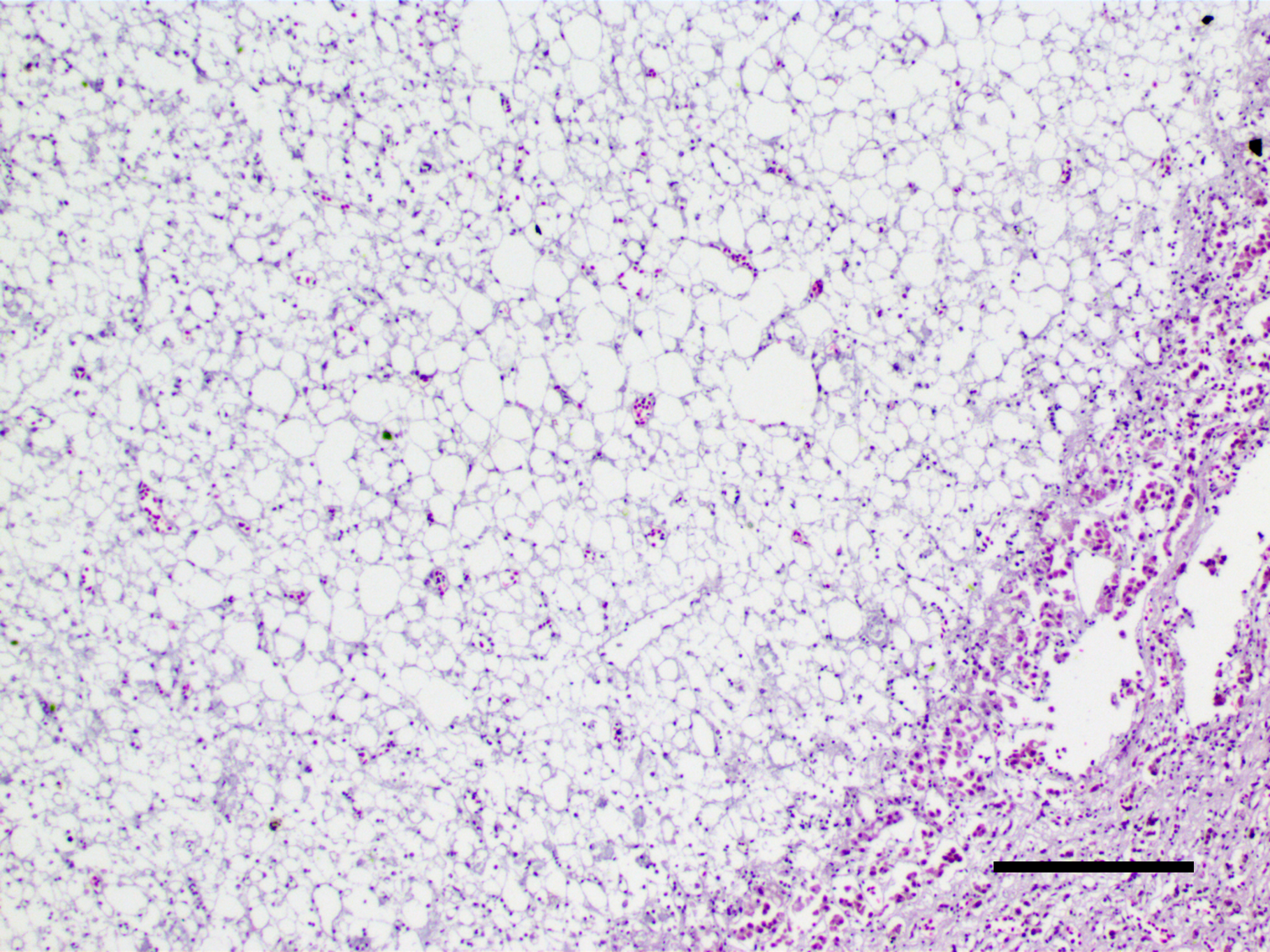

Supplement: Supplementary file 1 [file vetsci-10-00626-s001.zip › Fig. S1.tif]

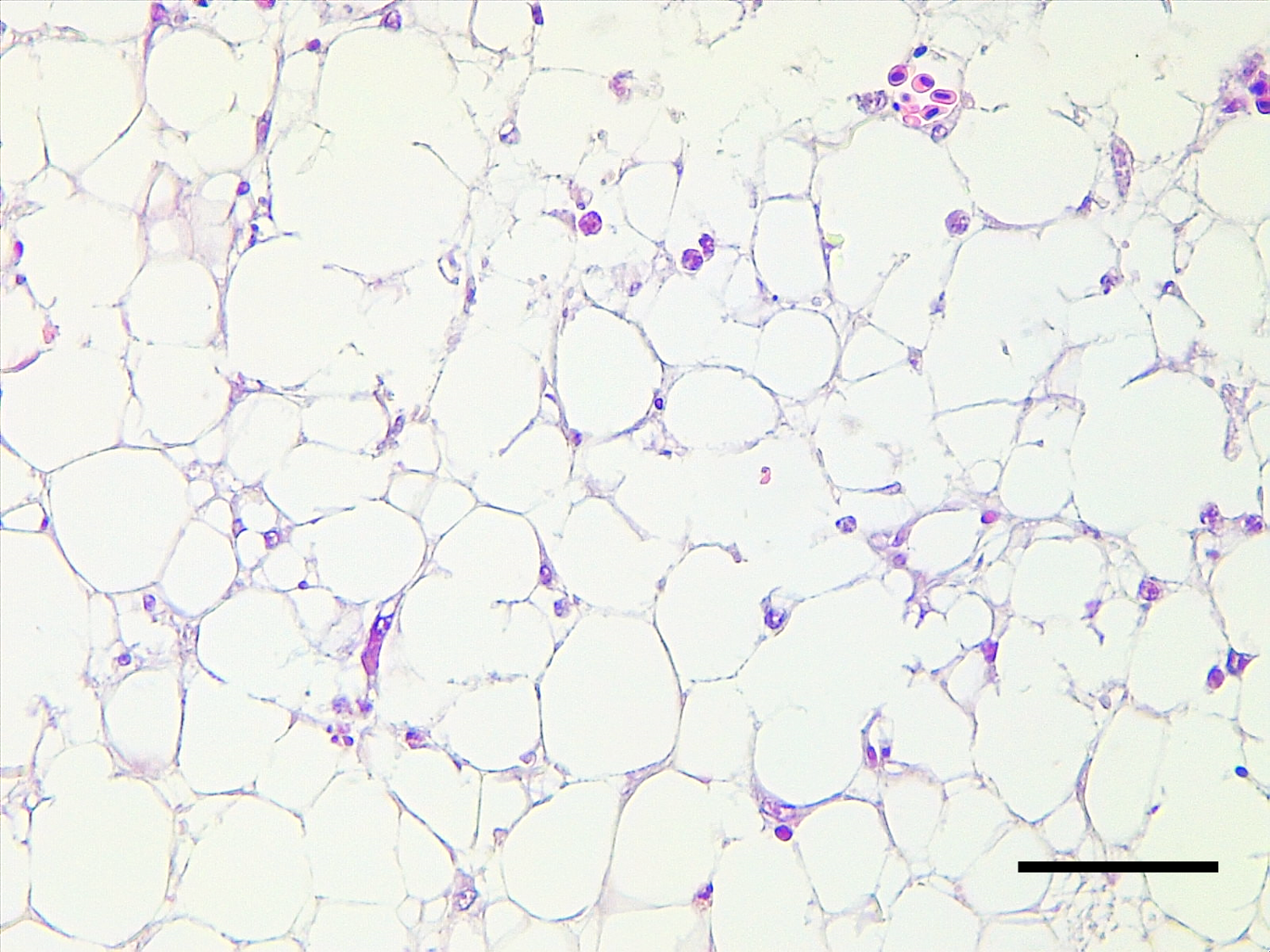

Supplement: Supplementary file 1 [file vetsci-10-00626-s001.zip › Fig. S2.tif]

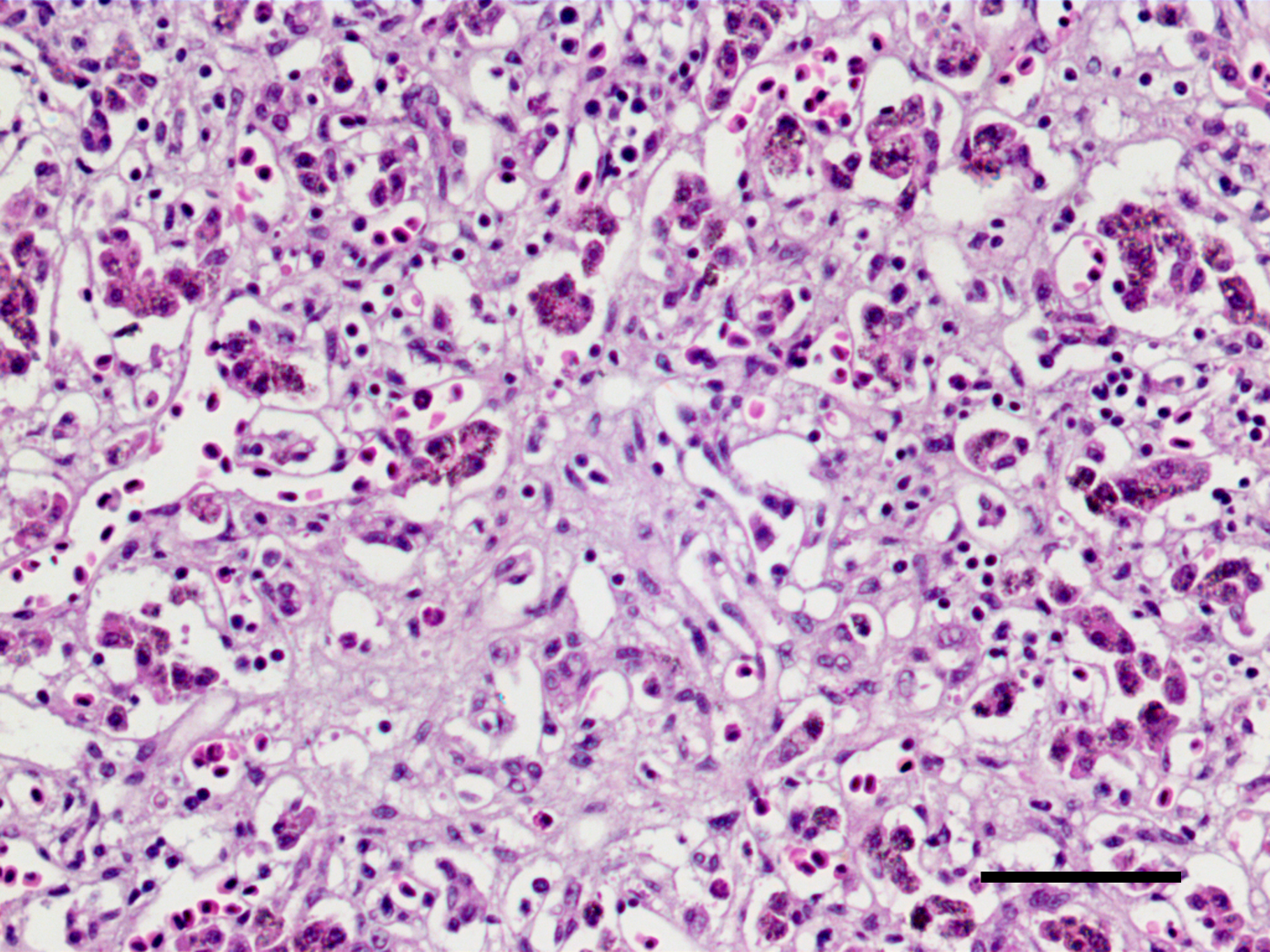

Supplement: Supplementary file 1 [file vetsci-10-00626-s001.zip › Fig. S3.tif]

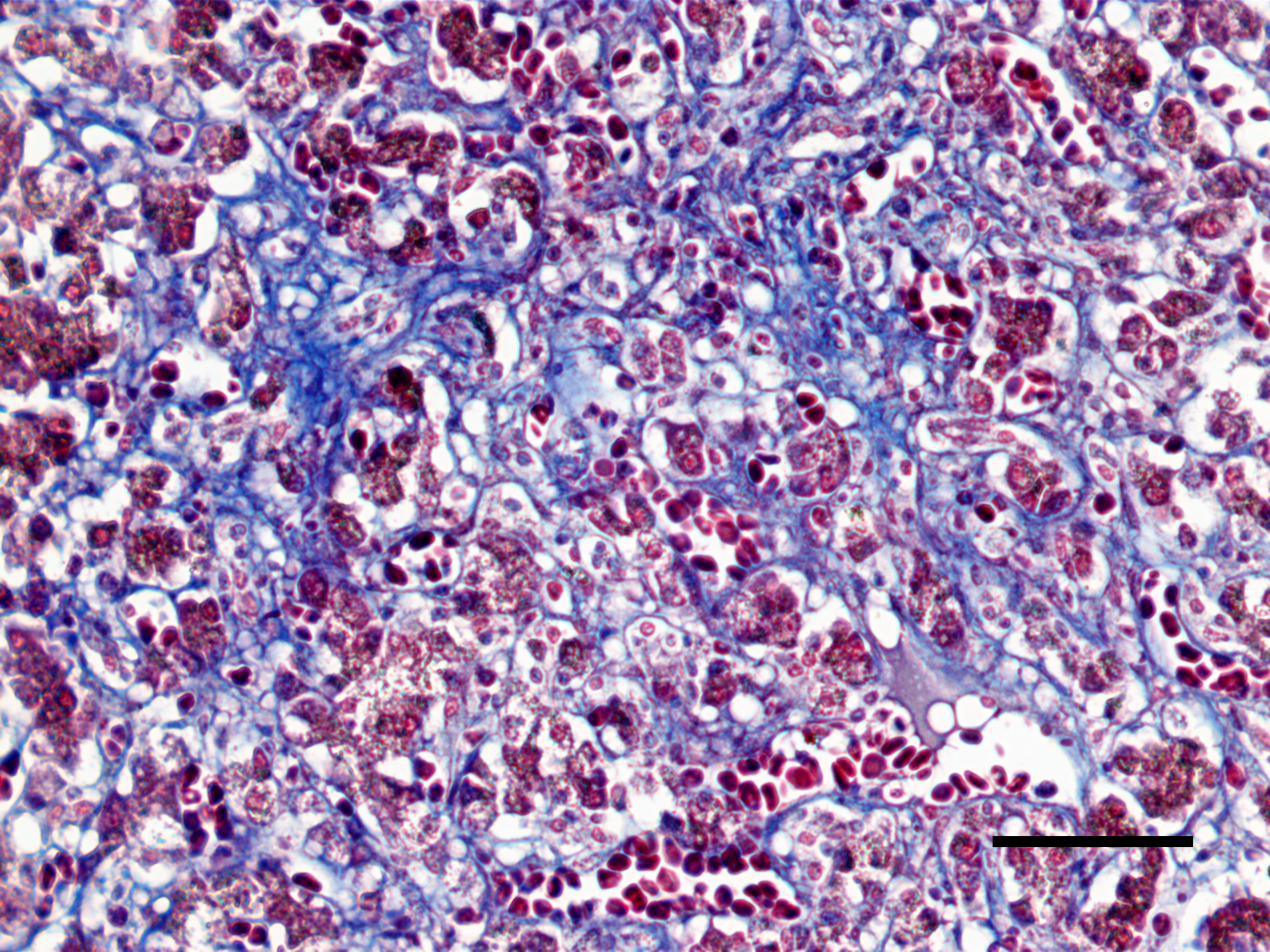

Supplement: Supplementary file 1 [file vetsci-10-00626-s001.zip › Fig. S4.tif]

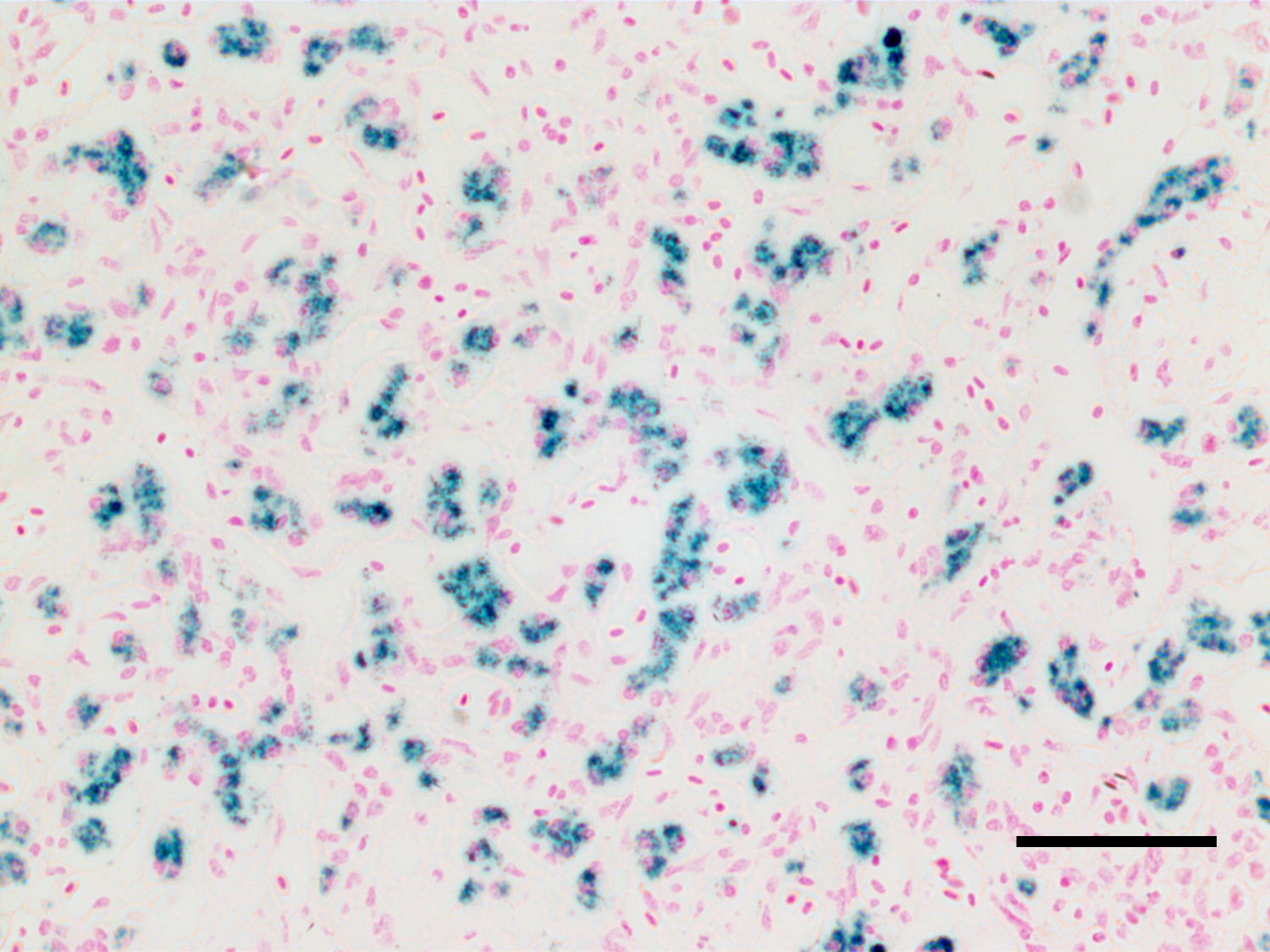

Supplement: Supplementary file 1 [file vetsci-10-00626-s001.zip › Fig. S5.tif]

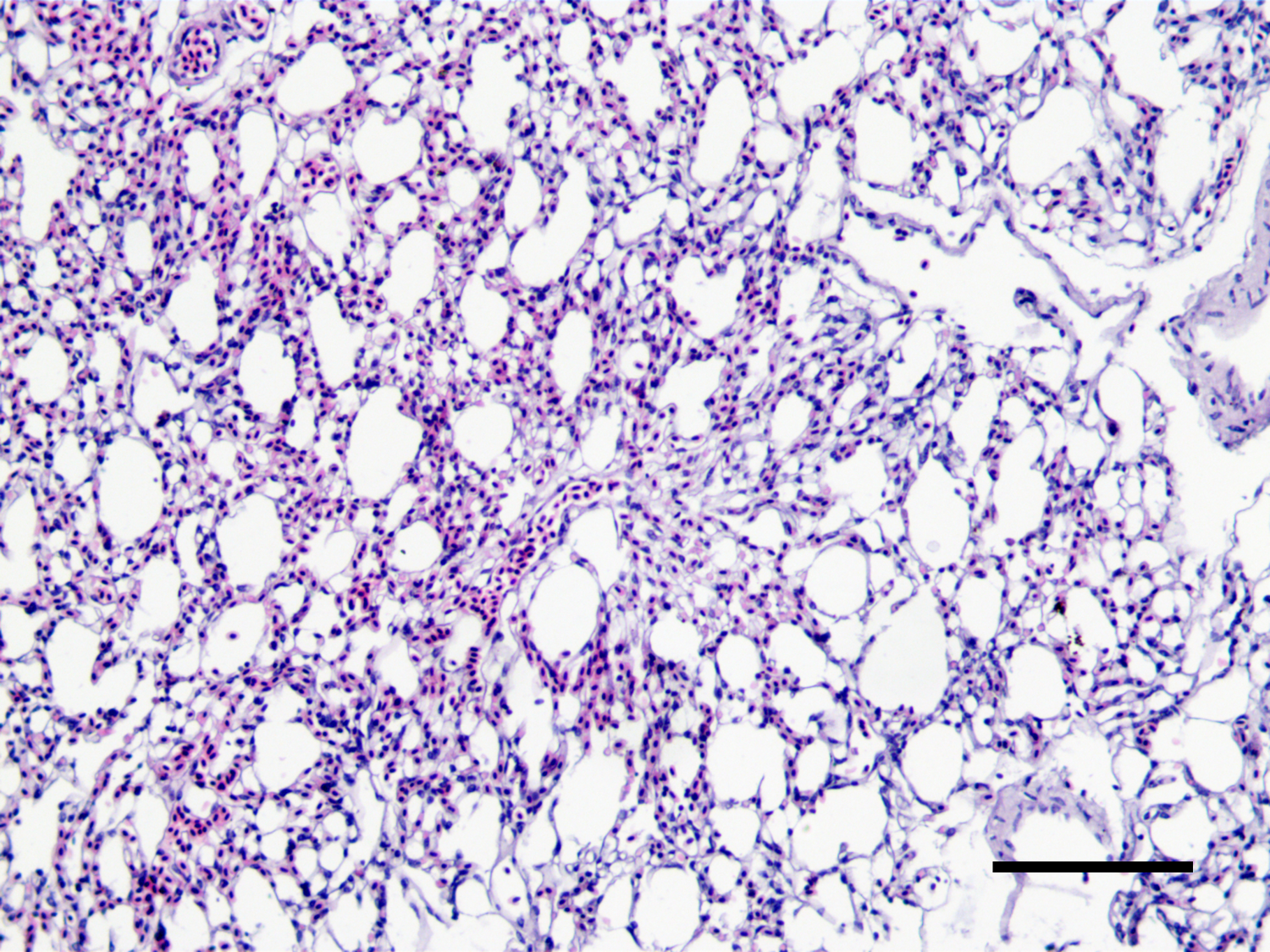

Supplement: Supplementary file 1 [file vetsci-10-00626-s001.zip › Fig. S6.tif]

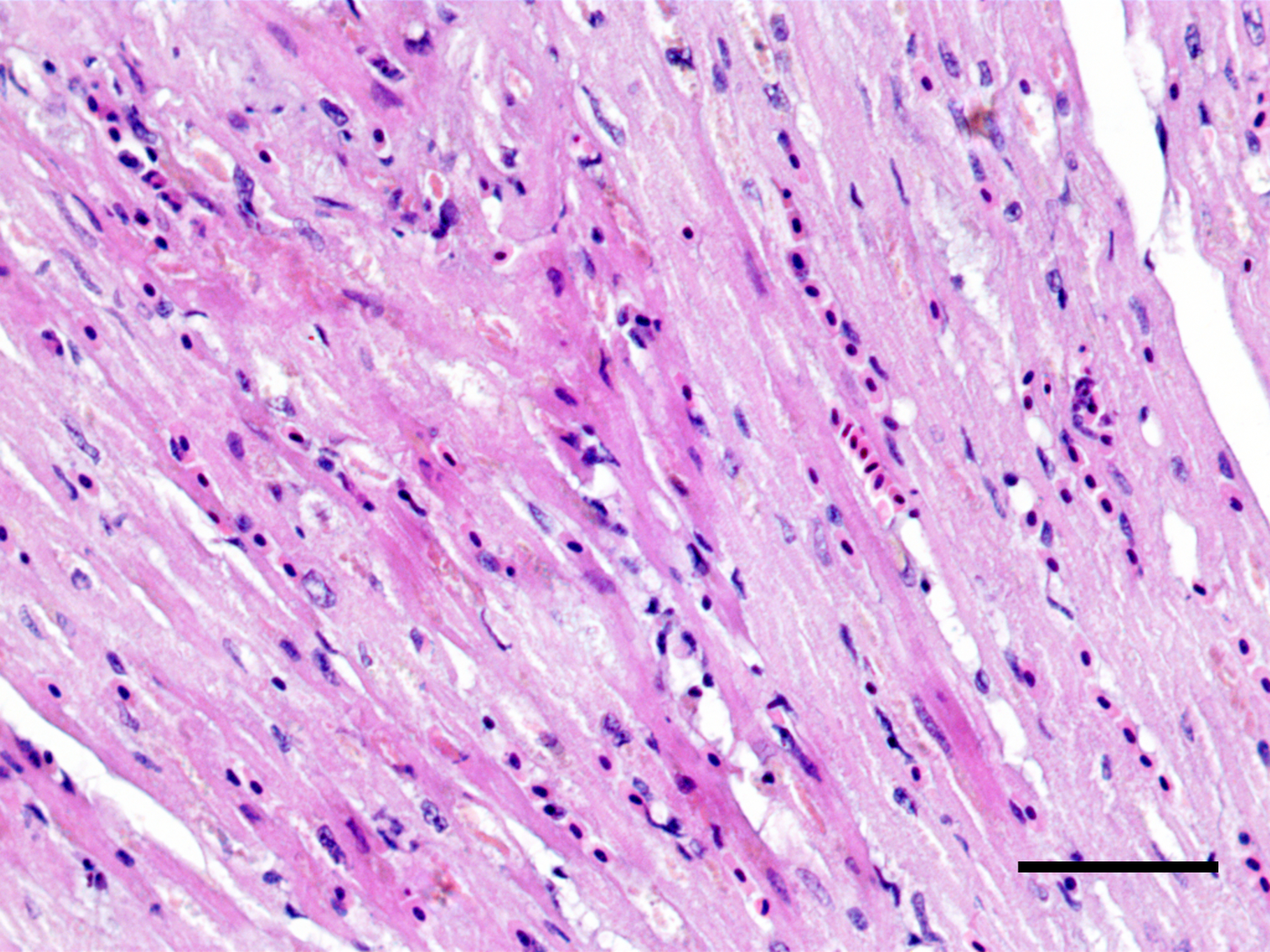

Supplement: Supplementary file 1 [file vetsci-10-00626-s001.zip › Fig. S7.tif]

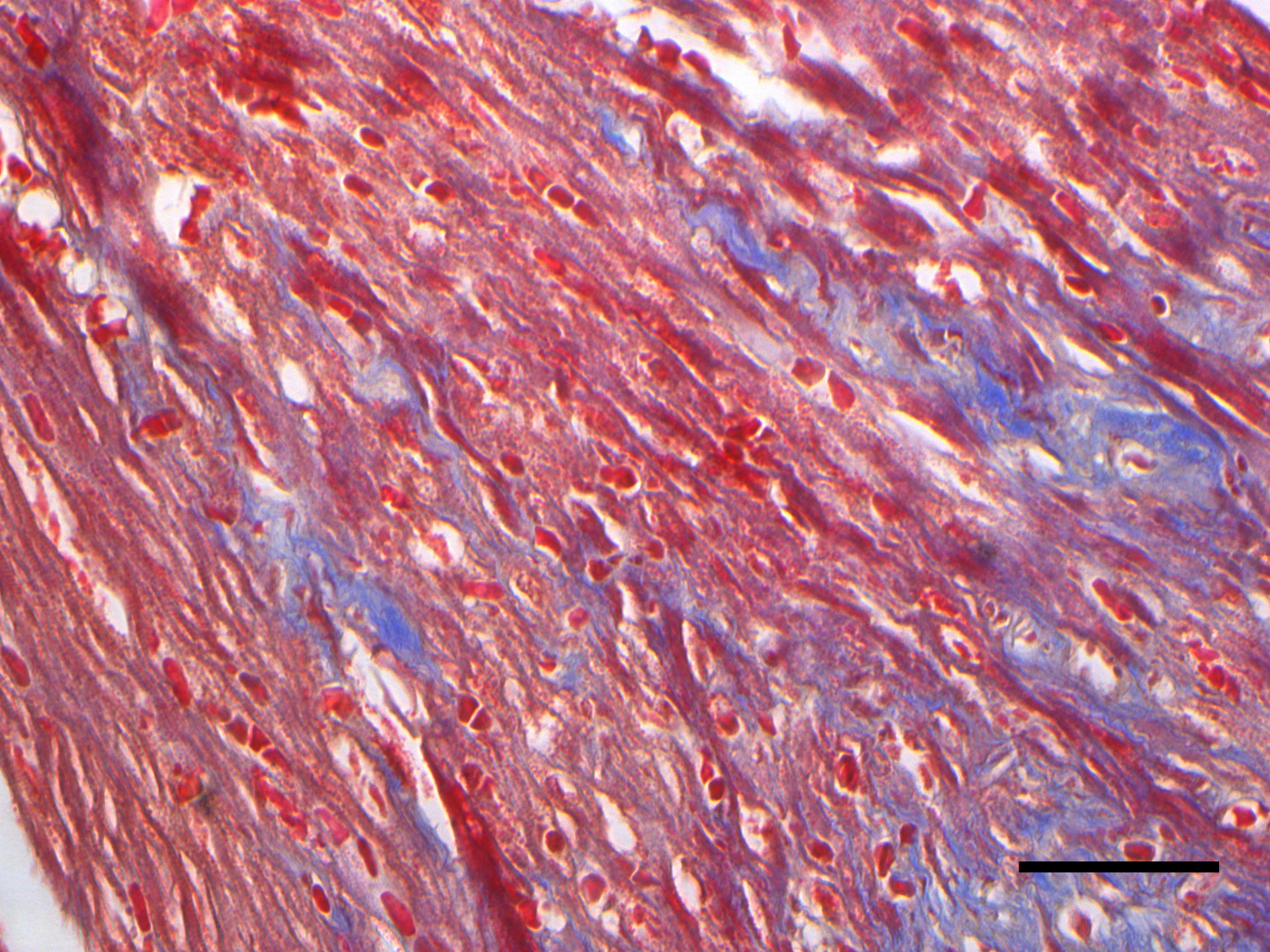

Supplement: Supplementary file 1 [file vetsci-10-00626-s001.zip › Fig. S8.tif]
